# Effect of Exposure Angulation on the Occupational Radiation Exposure during Cardiac Angiography: Simulation Study

**DOI:** 10.3390/ijerph18158097

**Published:** 2021-07-30

**Authors:** Younghoon Roh, Jungsu Kim, Hyemin Park, Jungmin Kim, Dongryeol Ryu, Kwangjin Chun, Jeonghun Seo, Bongki Lee, Byungryul Cho, Yongsu Yoon

**Affiliations:** 1 Department of Health and Safety Convergence Sciences, Korea University, 145 Anam-ro, Seongbuk-gu, Seoul 02841, Korea; dkviolet87@gmail.com (Y.R.); joae12@korea.ac.kr (H.P.); minbogun@korea.ac.kr (J.K.); 2 Department of Radiologic-Technology, Daegu Health College, 15, Yeongsong-ro, Buk-gu, Daegu 41453, Korea; rtkjs@korea.ac.kr; 3 Division of Cardiology, Department of Internal Medicine, Kangwon National University Hospital, Kangwon National University School of Medicine, Kangwon National University, Baengnyeong-ro 156, Chuncheon-si 24289, Korea; rdr0203@hanmail.net (D.R.); imchunn@naver.com (K.C.); hunsj@naver.com (J.S.); mdbklee@kangwon.ac.kr (B.L.); 4 Department of Radiological Science, Dongseo University, 47 Jurye-ro, Sasang-gu, Busan 47011, Korea

**Keywords:** occupational radiation exposure, cardiac angiography, Monte Carlo simulation, organ-dose

## Abstract

Cardiac angiography to visualize the cardiac coronary artery for lesions causes a lot of radiation exposure dose to the interventional cardiologist. We evaluated the occupational radiation exposure to the interventional cardiologist based on changes to the angle of the X-ray tube used in cardiac angiography and calculated the conversion factor for effective dose in this study. To evaluate the occupational radiation exposure resulting from scattered radiation to interventional cardiologists, organ doses for eyeball, thyroid, and heart were calculated using Monte Carlo simulation with korean typical man(KTMAN) phantom at the left anterior oblique (LAO)30/cranial (CRAN)30, CRAN40, right anterior oblique (RAO)30/CRAN30, RAO30/caudal(CAUD)20, CAUD39, LAO40/CAUD35, and LAO40 positions in the femoral and the radial artery puncture. In this study, analysis of the different angles showed the highest radiation exposure on LAO30/CRAN30 and CRAN40 position, which were 150.65% and 135.3%, respectively, compared to AP angles. Therefore, to reduce occupational dose for interventional cardiologists, it is recommended that radiation protection, such as using radiation shield and personal protective equipment (PPE), be used at LAO30/CRAN30 and CRAN40 angulation, and the conversion factor for calculating the organ dose received by the interventional cardiologists based on patient dose can be applied for improved occupational dose management.

## 1. Introduction

Since their discovery by Roentgen in 1895, X-rays have proven to be an essential diagnostic tool. A key component of radiology, X-rays are also employed in many other medical fields. For example, cardiac angiography and interventional procedures apply X-rays in the diagnosis and therapeutic procedures pertaining to cardiovascular diseases [1]. Coronary angiography is a technique used to visualize the coronary artery via radiation fluoroscopy and exposure techniques after contrast agents have been injected via a catheter through the femoral or radial arteries [2]. In coronary angiography, in which the patient lies supine on the examination table, the X-ray tube moves rotationally in two perpendicular planes, thereby enabling projection flexibility [3]. Such procedures inevitably subject patients and medical staff to radiation exposure. Although exposing patients to medical radiation does not impose restrictions, occupational radiation exposure for medical staff is not permitted to exceed an effective dose limit of 20 mSv per year, averaged over a 5 years period, and cannot exceed 50 mSv in a single year [4]. Therefore, occupational exposure to interventional cardiology staff is a critical issue in ensuring safe working conditions for medical professionals [5,6,7,8,9]. In order to observe the three large cardiac vessels of the heart, cardiac angiography acquires images via a combination of fluoroscopy and multi-angle X-ray exposure. In general, the shape of the cardiovascular system is checked according to the LAO/CRAN, CRAN, RAO/CRAN, RAO/CAUD, and CAUD views. Specifically, LAO/CAUD is the basis for judging cardiac lesions. Therefore, we evaluated the occupational radiation exposure for interventional cardiologists according to the angle of the X-ray tube used in cardiac angiography.

## 2. Materials and Methods

To evaluate the occupational radiation exposure resulting from scattered radiation for interventional cardiologists, we performed Monte Carlo simulations and a phantom study. Monte Carlo simulations were performed using the MCNPX V2.7 (Los Alamos National Laboratory, Los Alamos, NM, USA), which is an all particle, all energy Monte Carlo transport model. To determine representative values of the occupational radiation exposure, the occupational organ-absorbed dose for the eyes, thyroid, and heart were evaluated based on a single coronary angiography procedure at seven angulations. The organ-absorbed dose for the eyes, from the left and right entire eye, were averaged including cornea, lens glass, retinal, etc. Angiography was performed using an Allura Xper FD20 X-ray system (Philips, Amsterdam City, The Netherlands), with the relevant parameters incorporated into the Monte Carlo simulation. The X-ray simulation code SRS 78 (Institute of Physics and Engineering in Medicine, York, UK) was used to obtain a continuous X-ray spectrum. In this simulation, the absorbed organ dose was considered for an operator both wearing and not wearing personal protective equipment (PPE) by using the *F6 tally (i.e., the average energy-deposition over a cell [MeV/g]). The energy cutoff was 50 eV and the number of histories was checked to ensure a statistical error below 5%. In addition, an experimental measurement using a phantom was conducted to obtain the patient dose under equivalent conditions to the simulation. The patient dose was extracted using dose report data issued in angiography [10].

A. Simulation geometry

**Figure 1:**
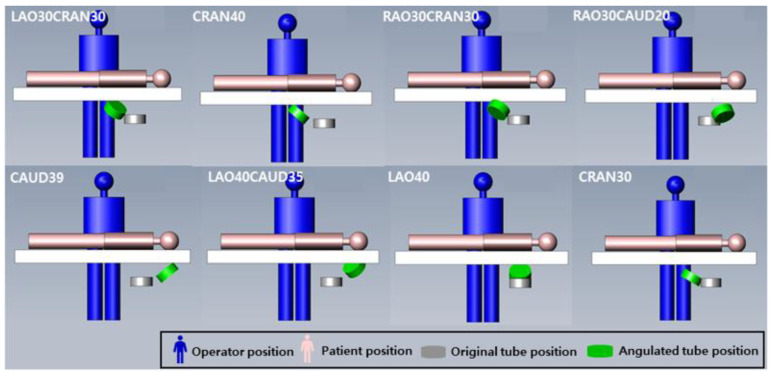
The simulated arrangement of interventional cardiologists and patients for cardiac angiography to X-ray angulation. The position of cardiologist, patient, and X-ray tube angulation showed in each procedure.

The geometric structure was modeled in accordance with the Allura Xper FD 20 X-ray system. Specifically, the dimensions of the patient table were set to 319 × 50 cm^2^ and the iso-center-to-floor distance was 113.5 cm. The fluoroscopy and X-ray exposure cine modes of the angiography device were both set at 15 fps. X-rays were generated at 64 kV with 0.7- and 0.4-mm Al-equivalent filtration, and the source-to-detector distance (SDD) was 119 cm. The rotation and angulation of the X-ray tube were set according to the conditions listed (shown) in Table 1 (Figure 1). The simulations were performed based on clinical research, while the study protocol was approved by the institutional ethics review committees of the respective study centers.

B. Anthropomorphic phantom for simulation study

**Figure 2:**
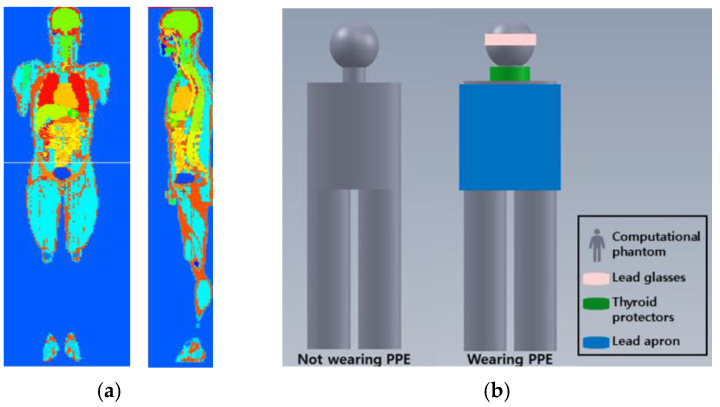
Computational phantom for Monte Carlo simulation. (**a**) Screenshot of Korean typical man-2 (KTMAN, sagittal and coronal plane); (**b**) the arrangement of the personal protective equipment (PPE) in the phantom. Lead glasses, thyroid protectors, and lead apron are located at eye, thyroid, and upper body.

Several anthropomorphic phantoms have been developed using Monte Carlo simulations. For example, KTMAN-2 is an anthropomorphic phantom based on computed tomography (CT) projections and was constructed using CT images of adult male volunteers corresponding to the reference data for Korean males (height: 172 cm, weight: 68 kg). KTMAN-2 consists of 300 × 150 × 344 voxels (each with a size of 2 × 2 × 5 mm^3^) and is divided into 48 anatomical regions [11]. Figure 2a shows the outline of the KTMAN-2 phantom. The phantom was used to evaluate the occupational organ doses for each procedure and was set in the position of the operator.

C. Personal protective equipment

The geometric and material properties of lead glasses, thyroid protectors, and lead aprons were included in the simulation: 0.75 mm-thick Pb, 0.5 mm-thick Pb, and 0.25 mm-thick Pb, respectively. The arrangement of the PPE for the phantom is shown in Figure 2b. The occupational dose was evaluated with and without PPE.

D. Measurements using the phantom

The whole-body phantom PBU-50 (Kyoto Kagaku, Japan) was constructed from human-equivalent materials and was placed in the standard position of a patient undergoing an angiography procedure. To verify the dose area product and air kerma for the procedure, the radiographic conditions were set as follows: fluoroscopy, 64 kV with 0.7-mm Al filtration, 4.58 mA, 8 shots lasting 30 s each; X-ray exposure, 64 kV with 0.4-mm Al filtration, 72.38 mA, 8 shots lasting 4 s each, 16 × 16 cm^2^ field of view. The nominal focal spot sizes of the fluoroscopy and X-ray exposure cine modes were 0.7 and 0.4 mm, respectively. The rotation and angulation of the X-ray tube were set using the same conditions as the simulation.

## 3. Results

The anthropomorphic phantom was positioned at the operator and patient positions according to the catheter insertion positions (radial and femoral artery) used for angiography, and the absorbed organ doses of the eye, thyroid gland, and heart were simulated for the operator according to the X-ray tube angle. The results are listed in Table 2. With PPE, the organ-absorbed doses of the eye, thyroid, and heart in the femoral artery puncture position (at 15 fps) were 90.32%, 86.96%, and 91.67%, respectively, of the corresponding radial artery puncture position doses. Without PPE, the organ-absorbed doses of the eye, thyroid gland, and heart in the femoral artery puncture position (at 15 fps) were 90.11%, 94.00%, and 91.18%, respectively, of the corresponding radial artery puncture position. The occupational organ absorbed doses exposed during angiography were not significantly different according to puncture location.

The organ-absorbed doses according to the exposure angle for the eye, thyroid gland, and myocardium of the heart are listed in Table 3. All results were calculated with and without PPE. The organ-absorbed doses according to puncture location (femoral and radial artery) were averaged. When the organ-absorbed doses acquired at different irradiation angles were normalized to the reference measurement at zero degrees, the occupational exposure dose increased for the RAO/CAUD position. The doses absorbed by the heart at the LAO30/CRAN30, CRAN40, RAO30/CRAN30, RAO30/CAUD20, CAUD 39, LAO40/CAUD35, and LAO40 positions for fluoroscopy at non-wearing PPE conditions were 150.65%, 135.30%, 90.23%, 79.22%, 84.38%, 86.30%, and 108.85% of the absorbed dose at the zero-degree reference position, respectively. For wearing PPE, the corresponding doses were 150.64%, 135.54%, 90.24%, 79.19%, 85.27%, 85.82%, and 108.84% of the absorbed dose at the zero degrees reference position. The normalized angle-resolved organ-absorbed doses for the eye, thyroid gland, and myocardium of the heart are presented in Table 4.

When replicating the X-ray exposure conditions used for the simulation, the dose area product and air kerma of the angiography device increased in proportion to the X-ray exposure conditions (i.e., tube voltage, current, and exposure time). The results of the dose area product and air kerma are listed in Table 5.

## 4. Discussion

Factors affecting the occupational dose in cardiology include frame rate, image amplification, beam collimation, procedure time, and diagnostic and working projection [12]. Analyzing the simulated occupational organ-absorbed dose reveals that, in comparison with the radial artery puncture procedure, the average doses received during the femoral artery puncture procedure were reduced by 8.85% and 9.75% with and without PPE, respectively. Depending on the intubation location, the distances between the operator and the patient in the femoral and radial artery puncture procedures were 120 cm and 100 cm, respectively, with the reduction in radiation exposure by distance calculated as 30.5%. The difference in the occupational dose according to the puncture position calculated by the simulation study was less than that calculated by simply considering the difference in distance, indicating that the location of the intubation does not have a significant impact on the occupational dose. Moreover, these results indicate that treatment time, which can vary according to the proficiency of the operator, is a major factor in determining the occupational dose assuming equivalent equipment and clinical environment [13].

The simulated absorbed organ dose was highest for the thyroid gland, followed by the eye, then the heart, indicating that the head of the operator is more vulnerable to scattered radiation than other body parts. Previous studies have reported increased instances of brain and neck cancer in operators performing intervention surgery relative to surgeons performing other procedures. These studies show that instances of brain cancer in operators performing interventional procedures are concentrated in the left side of the brain, with this imbalance suggesting a causal relationship to occupational radiation exposure, as the left side of the head receives greater exposure to scattered radiation than the right side during interventional procedures [14,15,16].

In terms of irradiation angle, both puncture procedures showed an identical trend, with the organ-absorbed dose highest for the LAO30/CRAN30 position and decreasing successively for the CRAN40, LAO40, RAO30/CRAN30, LAO40/CAUD35, CAUD39, and RAO30/CAUD20 positions. The major gantry angles that increased the occupational dose were the LAO and CRAN positions; in studies using dose-area products, such as that by Kuon et al., the increase of scattered radiation according to the irradiation angle was the same trend, tending to be highest at the LAO angle, with no significant difference reported between the cranial and caudal angles [17]. These results indicate that the occupational dose could be affected by reducing treatment times at critical angles, such as the LAO, which could achieve a significant dose reduction effect relative to other angles. The dose reduction for the thyroid gland, eye, and heart at the LAO30/CRAN30 angle can be higher by factors of 1.87, 15.7, and 1.80, respectively, relative to the CAUD39 angle when comparing femoral and radial artery puncture procedures.

We simulated the specific angiography device, Philips Allura Xper FD20, and the copper filtration was not implanted in the device. The low-energy scattered X-ray is a major factor for increasing patient dose [18]. When filtration for low-energy X-ray is added, the occupational dose can be reduced by reducing scattered radiation [19].

In this study, the occupational dose was calculated with the angles and the exposure condition used the coronary angiography (CAG) procedures. The CAG procedures using radiation have found widespread application in the diagnosis of cardiovascular disease with increased incidence of chronic disease. The coronary lesions found by CAG procedures are treated with percutaneous coronary intervention (PCI) procedures by inserting balloon and stent and the radiation exposure time is depending on the location or severity of the lesion [20]. The PCI procedures are an extension of the CAG procedures, and the angular occupational dose is applicable to the PCI procedures.

The latest angiography systems provide the dose area product and air kerma, allowing the operator to estimate the patient dose indirectly, with the patient information saved using DICOM. The relationship between air kerma and the dose absorbed by the patient has already been proven by calculating the conversion factor through phantom studies [21,22]. We derived a conversion factor for the angular dependency of the occupational dose using the air kerma value provided by the dose report (see Table 6). Consequently, we can check the occupational dose by calculating the patient dose and conversion factor and develop an occupational radiation management system with patient doses stored in DICOM.

## 5. Conclusions

Occupational radiation exposure is equally important as the patient dose in cardiac intervention. In cardiac interventional procedures, the occupational dose stems from scattered radiation, and it can be restricted via a combination of wearing PPE, minimizing the operating time, and careful irradiation angle selection [23,24]. In this study, the sensitivity of the occupational dose to the intubation location and irradiation angle were investigated, with limiting the procedure time at critical irradiation angles, such as LAO, shown to be the most effective method for reducing the exposure to scattered radiation. Significantly, we derived a conversion factor for calculating the dose received by the operator according to the dose delivered to the patient, which can be applied for improved occupational dose management.

## Figures and Tables

**Table 1:** The cardiac angiography exposure condition for Monte Carlo simulation and phantom study. X-ray tube rotation and angulation, distance from X-ray source to image detector, and exposure time showed in each procedure.

Mode	Rotation (Degree)	Angulation (Degree)	Source to Image Distance (cm)	Time (s)
FS ^(1)^	0	0	119	30
CA ^(2)^	LAO 30	CRAN 30	119	4
FS	LAO 30	CRAN 30	119	30
CA	0	CRAN 40	119	4
FS	0	CRAN 40	119	30
CA	RAO 30	CRAN 30	119	4
FS	RAO 30	CRAN 30	119	30
CA	RAO 30	CAUD 20	119	4
FS	RAO 30	CAUD 20	119	30
CA	0	CAUD 39	119	4
FS	0	CAUD 39	119	30
CA	LAO 40	CAUD 35	119	4
FS	LAO 40	CAUD 35	119	30
CA	LAO 40	0	119	4
FS	LAO 40	0	119	30
CA	0	CRAN 30	119	4

^(1)^ Fluoroscopy, ^(2)^ Cine angiography.

**Table 2:** Monte Carlo N-particle code-simulation results of each exposure condition for the organ doses of the eyeball, thyroid, and heart of cardiac operator.

Mode	Puncture Position	Protection Device	Eyeball (mGy)	Thyroid (mGy)	Heart (mGy)
(Frames/s)	(cm)		Uncertainty (%)	Uncertainty (%)	Uncertainty (%)
15	Radial artery 100	Used	3.10E-04 0.40	4.60E-04 0.40	1.20E-04 0.40
		Non used	9.10E-03 0.7	5.00E-03 0.7	3.40E-03 0.7
	Femoral artery 120	Used	2.80E-04 0.7	4.00E-04 0.7	1.10E-04 0.7
		Non used	8.20E-03 0.6	4.70E-03 0.6	3.10E-03 0.6

**Table 3:** The simulated occupational organ dose for cardiac angiography with and without PPE.

Mode	Rotation (Degree)	Angulation (Degree)	Average Radiation Dose Non-Wearing PPE (mSv)			Average Radiation Dose Wearing PPE (mSv)		
			Thyroid	Eye	Heart	Thyroid	Eye	Heart
FS	0	0	8.16E-04	4.95E-04	2.72E-04	4.11E-05	3.08E-05	9.52E-06
CA	LAO 30	CRAN 30	4.63E-04	3.38E-04	1.84E-04	2.39E-05	1.93E-05	6.68E-06
FS	LAO 30	CRAN 30	1.03E-03	7.41E-04	4.09E-04	5.21E-05	4.62E-05	1.44E-05
CA	0	CRAN 40	2.38E-04	1.55E-04	9.42E-05	1.33E-05	1.05E-05	3.60E-06
FS	0	CRAN 40	1.02E-03	6.84E-04	3.68E-04	4.78E-05	3.94E-05	1.22E-05
CA	RAO 30	CRAN 30	3.16E-04	1.81E-04	1.11E-04	1.59E-05	1.09E-05	3.85E-06
FS	RAO 30	CRAN 30	7.03E-04	3.97E-04	2.45E-04	3.55E-05	2.49E-05	8.56E-06
CA	RAO 30	CAUD 20	2.56E-04	3.28E-05	9.68E-05	1.44E-05	1.33E-06	3.74E-06
FS	RAO 30	CAUD 20	5.75E-04	7.33E-05	2.15E-04	2.90E-05	2.68E-06	7.63E-06
CA	0	CAUD 39	2.60E-04	2.21E-05	1.04E-04	1.21E-05	1.17E-06	3.37E-06
FS	0	CAUD 39	5.76E-04	4.83E-05	2.32E-04	1.98E-05	2.06E-06	5.63E-06
CA	LAO 40	CAUD 35	2.56E-04	1.82E-04	1.05E-04	1.29E-05	1.09E-05	3.64E-06
FS	LAO 40	CAUD 35	5.74E-04	3.99E-04	2.33E-04	3.43E-05	2.94E-05	9.52E-06
CA	LAO 40	0	3.35E-04	2.47E-04	1.33E-04	2.02E-05	1.55E-05	5.05E-06
FS	LAO 40	0	7.44E-04	5.42E-04	2.96E-04	3.75E-05	3.39E-05	1.04E-05
CA	0	CRAN 30	4.58E-04	3.10E-04	1.65E-04	2.14E-05	1.79E-05	5.96E-06
Simulated organ dose			8.62E-03	4.85E-03	3.26E-03	4.31E-04	2.97E-04	1.14E-04

**Table 4:** The simulated occupational organ dose ratio according to rotation and angulation degree of the X-ray tube (zero-degree angle reference) with and without PPE condition.

Mode	Rotation (Degree)	Angulation (Degree)	Average Radiation Dose Non-Wearing PPE (mSv)			Average Radiation Dose Wearing PPE (mSv)		
			Thyroid	Eye	Heart	Thyroid	Eye	Heart
FS	0	0	100.00%	100.00%	100.00%	100.00%	100.00%	100.00%
	LAO 30	CRAN 30	126.24%	149.70%	150.64%	126.92%	149.84%	150.81%
	0	CRAN 40	125.14%	138.18%	135.54%	116.32%	127.76%	128.22%
	RAO 30	CRAN 30	86.14%	80.10%	90.24%	86.36%	80.68%	89.91%
	RAO 30	CAUD 20	70.45%	14.80%	79.19%	70.52%	8.69%	80.19%
	0	CAUD 39	70.57%	9.76%	85.27%	48.11%	6.69%	59.12%
	LAO 40	CAUD 35	70.39%	80.61%	85.82%	83.43%	95.45%	100.00%
	LAO 40	0	91.23%	109.39%	108.84%	91.35%	110.06%	109.25%

**Table 5:** The dose-report for cardiac angiography.

Mode	Rotation (Degree)	Angulation (Degree)	Dose Area Product (Gy∙cm^2^)	Air Kerma (mGy)
FS	0	0	0.48	2.01
CA	LAO 30	CRAN 30	0.12	0.50
FS	LAO 30	CRAN 30	0.47	1.91
CA	0	CRAN 40	0.13	0.52
FS	0	CRAN 40	0.76	3.15
CA	RAO 30	CRAN 30	0.24	1.01
FS	RAO 30	CRAN 30	0.55	2.27
CA	RAO 30	CAUD 20	0.10	0.42
FS	RAO 30	CAUD 20	0.49	1.97
CA	0	CAUD 39	0.15	0.59
FS	0	CAUD 39	0.54	2.25
CA	LAO 40	CAUD 35	0.24	1.01
FS	LAO 40	CAUD 35	0.43	1.82
CA	LAO 40	0	0.09	0.39
FS	LAO 40	0	0.39	1.59
CA	0	CRAN30	0.10	0.42

**Table 6:** The conversion factor for patient air kerma to occupational organ dose in cardiac angiography.

Mode	Rotation (Degree)	Angulation (Degree)	Average Radiation Dose Non-Wearing PPE (mSv)			Average Radiation Dose Wearing PPE (mSv)		
			Thyroid	Eye	Heart	Thyroid	Eye	Heart
FS	0	0	4.06E-04	2.46E-04	1.35E-04	2.04E-05	1.53E-05	4.74E-06
CA	LAO 30	CRAN 30	9.26E-04	6.76E-04	3.68E-04	4.78E-05	3.86E-05	1.34E-05
FS	LAO 30	CRAN 30	5.39E-04	3.88E-04	2.14E-04	2.73E-05	2.42E-05	7.54E-06
CA	0	CRAN 40	4.58E-04	2.98E-04	1.81E-04	2.56E-05	2.02E-05	6.92E-06
FS	0	CRAN 40	3.24E-04	2.17E-04	1.17E-04	1.52E-05	1.25E-05	3.87E-06
CA	RAO 30	CRAN 30	3.13E-04	1.79E-04	1.10E-04	1.57E-05	1.08E-05	3.81E-06
FS	RAO 30	CRAN 30	3.10E-04	1.75E-04	1.08E-04	1.56E-05	1.10E-05	3.77E-06
CA	RAO 30	CAUD 20	6.10E-04	7.81E-05	2.30E-04	3.43E-05	3.17E-06	8.90E-06
FS	RAO 30	CAUD 20	2.92E-04	3.72E-05	1.09E-04	1.47E-05	1.36E-06	3.87E-06
CA	0	CAUD 39	4.41E-04	3.75E-05	1.76E-04	2.05E-05	1.98E-06	5.71E-06
FS	0	CAUD 39	2.56E-04	2.15E-05	1.03E-04	8.80E-06	9.16E-07	2.50E-06
CA	LAO 40	CAUD 35	2.53E-04	1.80E-04	1.04E-04	1.28E-05	1.08E-05	3.60E-06
FS	LAO 40	CAUD 35	3.15E-04	2.19E-04	1.28E-04	1.88E-05	1.62E-05	5.23E-06
CA	LAO 40	0	8.59E-04	6.33E-04	3.41E-04	5.18E-05	3.97E-05	1.29E-05
FS	LAO 40	0	4.68E-04	3.41E-04	1.86E-04	2.36E-05	2.13E-05	6.54E-06
CA	0	CRAN 30	1.09E-03	7.38E-04	3.93E-04	5.10E-05	4.26E-05	1.42E-05

## Data Availability

Data can be requested from the corresponding author.
